# Supplementary material for: A set of multi-entry identification keys to African frugivorous flies (Diptera, Tephritidae)
Source: Zookeys. 2014 Jul 24;(428):97–108. doi: 10.3897/zookeys.428.7366 (PMC4143993; doi:10.3897/zookeys.428.7366)
Supplement: Supplementary material 6 — Key to Ceratitis [file zookeys-428-097-s006.zip › SF6_ZooKeys_key to Ceratitis/key/SF6_ZooKeys_key to Ceratitis/Media/Html/Ceratitis brucei .htm]

Ceratitis (Ceratalaspis) brucei Munro


***Ceratitis*** **(*Ceratalaspis*)
*brucei* Munro**

*Ceratitis* (*Pardalaspis*) *brucei*
Munro, 1935: 308.

Body
length: 5.40 mm; wing length: 5.75 mm.

Female

Head.
Antenna yellow-orange. Antennal implant with a faint horizontal band (possibly
discolouration). Third antennal segment twice as long as second segment. Arista
missing in holotype. Frons with short scattered hairs which are largely the
same colour or sligthly darker than frons; convex, in lateral view not
distinctly projecting forwards at antennal implant. Chaetotaxy normal for
subgenus.

Thorax.
Postpronotum white, with black spot. Ground colour of mesonotum pale, with
orange tinge. Chaetotaxy normal for subgenus. Scapular setae black. One
anepisternal bristle. Scutellum white basally, otherwise yellow with three black
separate markings apically; basally with 2 dark, distinct spots. Subscutellum
completely dark.

Legs.
Yellow; setation typical for subgenus, mainly pale especially on femora.
Posterior and posterodorsal rows on fore femur pale.

Wing.
Bands with yellow-brown markings (yellow areas quite extensive but not
predominant). Banding, marginal band continuous; cubital band free; medial band
absent; crossvein r-m at middle of discal cell. Crossvein dm-cu posteriorly
more outwards than anteriorly.

Abdomen.
Pale yellow-brown. Setation and banding typical for subgenus. Oviscape almost
as long as abdominal terga 3-6 combined. Aculeus, tip in holotype exposed but
broken; not dissected but apparently straight and simple, without serration or
notches/appendices.

Male

Unknown.

(Description
after De Meyer, 1998)
